# Significance of Technetium-99m Human Serum Albumin Diethylenetriamine Pentaacetic Acid Scintigraphy in Patients with Nephrotic Syndrome

**DOI:** 10.1371/journal.pone.0123036

**Published:** 2015-04-10

**Authors:** Tsuyoshi Takashima, Tomoya Kishi, Koji Onozawa, Shuichi Rikitake, Motoaki Miyazono, Takateru Otsuka, Hiroyuki Irie, Ryuichi Iwakiri, Kazuma Fujimoto, Yuji Ikeda

**Affiliations:** 1 Department of Internal Medicine, Saga University Faculty of Medicine, Saga, Japan; 2 Department of Nephrology, Ureshino Medical Center, Ureshino, Japan; 3 Department of Radiology, Saga University Faculty of Medicine, Saga, Japan

## Abstract

It is thought that a large amount of albumin leaking from the glomerulus in nephrotic syndrome (NS) is reabsorbed at the proximal tubule and catabolized. Therefore, it is possible the final quantity of urinary protein does not always reflect the amount of leakage of protein from the glomerulus. We experienced two cases without nephrotic range proteinuria thought to involve hypoproteinemia due to the same pathophysiology as NS. On these patients, we performed protein leakage scintigraphy with technetium-99m human serum albumin diethylenetriamine pentaacetic acid (^99m^Tc-HSAD) to exclude a diagnosis of protein-losing gastroenteropathy and observed diffuse positive accumulation in the kidneys with more intense uptake in the kidney than the liver on the anterior view 24 hours after ^99m^Tc-HSAD administration. In healthy adults intravenously given ^99m^Tc-HSAD, the same dynamics are observed as in albumin metabolism, and the organ radioactivity of the liver and kidneys after 24 hours is equal. Therefore, we thought it was possible that the renal uptake 24 hours after ^99m^Tc-HSAD administration was a characteristic finding of NS. In order to confirm it, the subjects were divided into two groups: the NS group (n = 10) and the non-NS group (n = 7). We defined more intense uptake in the kidney than the liver on the anterior view 24 hours after ^99m^Tc-HSAD administration as Dense Kidney (+). Furthermore, we designed regions of interest in the right and left kidneys and liver on anterior and posterior images, then calculated the kidney-liver ratio. Nine of the ten patients had Dense Kidney (+) in the NS group, compared to none in the non-NS group. And the kidney-liver ratio was significantly higher in the NS group than in the non-NS group on each view in the bilateral kidneys. In conclusion, our results suggest that the renal uptake 24 hours after ^99m^Tc-HSAD administration is a characteristic finding of NS.

## Introduction

Many illness, including disturbances of protein synthesis (liver disease), inflammatory diseases and protein leakage from the gastrointestinal tract, can cause hypoproteinemia. Nephrotic syndrome (NS) is also a common cause, and the onset of hypoproteinemia is closely related to proteinuria, usually being diagnosed when the proteinuria is severe (generally more than 3.5 g/day). However, it has long been suggested that the degree of hypoproteinemia may not always depend on the severity of proteinuria alone [1]. Therefore, it is possible that the patients with NS exhibiting a the lack of nephrotic range proteinuria exist [2]. However, there have been no studies about such condition because of difficulty of a proof that it is the same condition as NS.

We previously reported two patients with minor glomerular abnormalities who did not exhibit a large enough quantity of urine protein to cause NS, although their hypoproteinemia responded to steroids [3]. We speculated that the pathophysiology was similar to that of minimal change NS because the renal pathology included foot process effacement and hyaline droplet degeneration, indicating hyperfunction of urine protein reabsorption at the proximal convoluted tubule due to massive leakage in the glomeruli, after excluding other diseases causing hypoproteinemia. At that time, protein leakage scintigraphy with technetium-99m human serum albumin diethylenetriamine pentaacetic acid (^99m^Tc-HSAD) was performed to exclude a diagnosis of protein-losing gastroenteropathy, the results of which showed diffuse positive accumulation in the kidneys with an uptake that was strikingly more intense than that in the liver on the anterior view 24 hours after ^99m^Tc-HSAD administration, and this was considered to be an uncommon finding.

We generally perform protein leakage scintigraphy with ^99m^Tc-HSAD to diagnose protein-losing gastroenteropathy. If there is the exudation of ^99m^Tc-HSAD from the gastrointestinal tract and the tracer visually accumulates in its region, protein leakage scintigraphy is considered positive.

**Fig 1:**
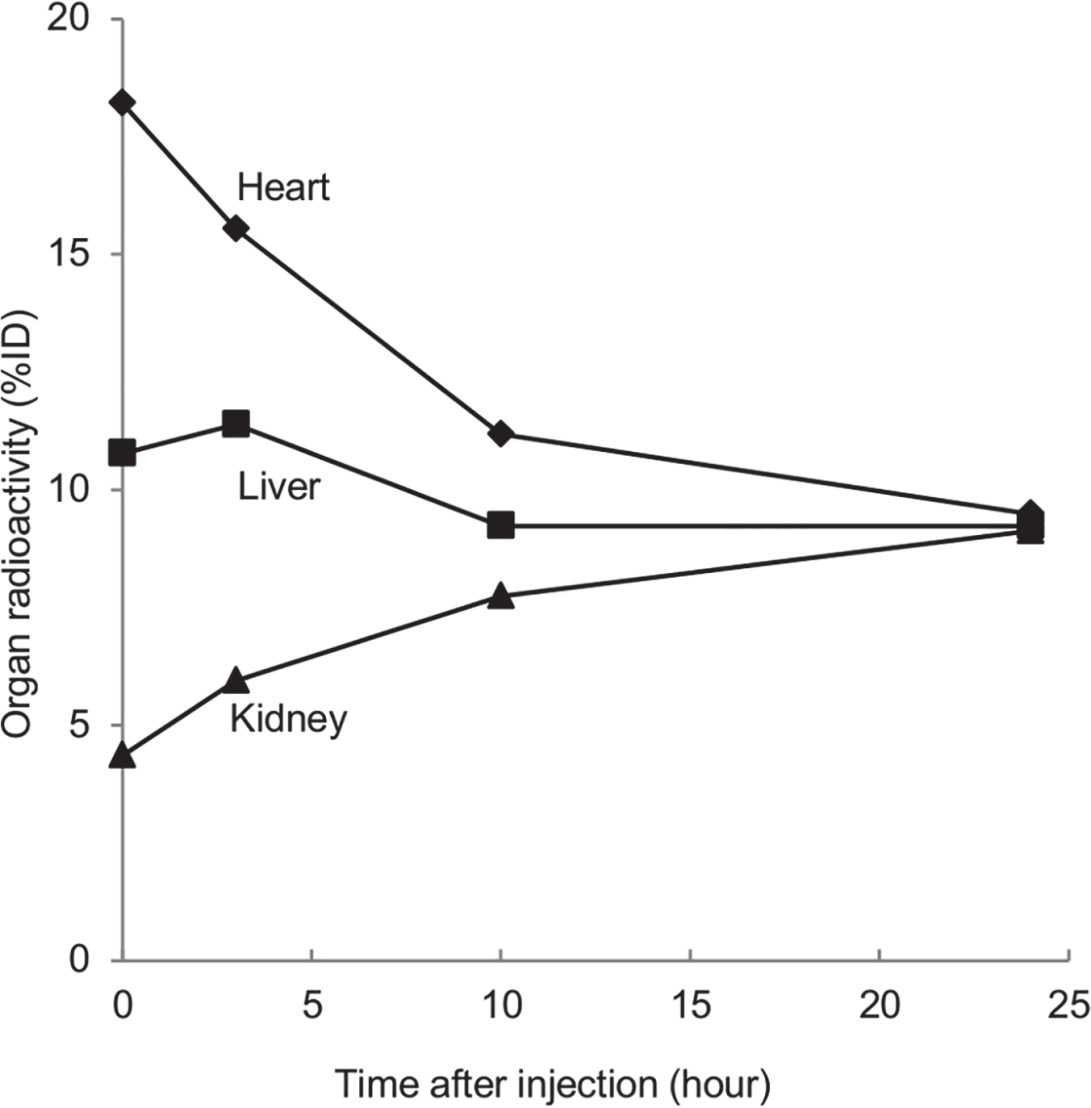
Sequential changes in the %ID in the main organs after the injection of ^99m^Tc-HSAD in a healthy adult. Reproduced from Tamaki N et al. [3]. Abbreviations. %ID: % injected dose.

In healthy adults intravenously given ^99m^Tc-HSAD for protein leakage scintigraphy, the material accumulates in bloodstream-rich organs (heart, liver, lungs, etc.), including the kidneys, according to the same dynamics as albumin metabolism, then continues to circulate through the blood vessels for a long period [4]. A previous study showed that the organ radioactivity (% injected dose: %ID) of the liver and kidneys after 24 hours in healthy adults is equal at approximately 10% (Fig 1) [5].

Therefore, the renal uptake 24 hours after ^99m^Tc-HSAD administration may suggest hyperfunction of urine protein reabsorption due to massive leakage from the glomeruli, which may be utilized to diagnose a nephrotic state. To the best of our knowledge, no similar studies have been reported to date. The objective of this study was to assess the efficacy of the renal uptake observed 24 hours after ^99m^Tc-HSAD administration to discriminate between NS and non-NS.

## Materials and Methods

### Ethics statement

This study was approved by the Human Research Ethics Committee of Saga University (Permit Number: 2013-07-10). The clinical data collected and analyzed in this study were obtained not for research purposes, but rather to provide appropriate medical care, and analyzed anonymously. This study was conducted according to the principles expressed in the Declaration of Helsinki.

### Study design

We performed a single-center, retrospective study of Japanese patients using medical records at the Saga University Faculty of Medicine, Saga, Japan. The study population included patients who were considered to need a differential diagnosis of protein leakage from the gastrointestinal tract, and who underwent protein leakage scintigraphy with ^99m^Tc-HSAD at our hospital between January 1, 2008 and March 31, 2014. A total of 33 patients received protein leakage scintigraphy with ^99m^Tc-HSAD. Subjects were excluded if they had no definitive diagnosis or urinary data. We defined the NS group (10 patients) as subjects with a urine protein-creatinine ratio (U-Pro/Cr) above 3.5 g/g and the non-NS group (seven patients) as those with a U-Pro/Cr below 0.3 g/g or equal findings from a qualitative analysis of urine (Table 1).

**Table 1:** Characteristics of 17 patients.

**NS group**	**Age (years)**	**Sex**	**BMI (kg/m^2^)**	**Proteinuria**	**U-pro/cr (g/g)**	**TP (g/dL)**	**Alb (g/dL)**	**T-cho (mg/dL)**	**LDL-C (mg/dL)**	**TG (mg/dL)**	**Cr (mg/dL)**	**disease**
**Case 1**	72	F	21.7	3+	6.06	5.1	2.2	270	191	159	0.58	membranous nephropathy
**Case 2**	33	F	20.2	4+	4.16	5.2	1.8	241	149	68	0.57	minimal change
**Case 3**	53	F	23.2	3+	3.60	5.2	2.7	228	145	88	0.86	minimal change
**Case 4**	76	F	18.4	4+	5.98	5.0	2.2	204	136	133	1.29	focal segmental glomerulosclerosis
**Case 5**	58	M	31.1	4+	5.43	3.4	1.0	383	279	190	1.68	focal segmental glomerulosclerosis
**Case 6**	75	F	21.1	4+	11.90	5.4	2.0	295	158	197	0.55	minimal change
**Case 7**	24	F	20.5	4+	4.51	4.3	0.9	433	279	171	0.66	minimal change
**Case 8**	57	F	21.9	4+	7.78	3.8	0.6	541	434	236	0.91	minimal change
**Case 9**	70	F	18.4	4+	6.12	3.9	1.4	377	281	261	0.53	membranous nephropathy
**Case 10**	70	F	21.8	4+	6.47	5.7	2.0	192	116	165	0.49	membranous nephropathy
**mean ± SD**	58.8 ± 17.9		21.8 ± 3.6		6.20 ± 2.34	4.70 ± 0.78	1.68 ± 0.68	316.4 ± 113.7	216.8 ± 99.8	166.8 ± 59.9	0.81 ± 0.39	
**non-NS group**	**Age**	**Sex**	**BMI**	**Proteinuria**	**U-pro/cr**	**TP**	**Alb**	**T-cho**	**LDL-C**	**TG**	**Cr**	**disease**
**Case 11**	79	F	27.7	(-)	ND	4.5	1.2	101	36	47	1.08	malabsorption syndrome, chronic pancreatitis
**Case 12**	70	M	17.0	(±)	0.18	5.7	2.4	143	89	107	0.83	malabsorption syndrome, chronic pancreatitis
**Case 13**	53	F	19.3	(-)	0.15	3.9	1.4	120	51	195	0.97	Crohn's disease, protein-losing gastroenteropathy
**Case 14**	59	M	18.7	(-)	ND	3.7	1.5	111	ND	152	0.53	eosinophilic gastroenteritis, protein-losing gastroenteropathy
**Case 15**	58	M	21.4	(-)	ND	6.4	3.4	197	117	171	0.59	Cronkhite-Canada syndrome
**Case 16**	14	F	22.5	(-)	ND	6.2	3.1	258	ND	229	0.54	Lupus entelitis
**Case 17**	72	M	17.7	(-)	ND	5.3	2.6	122	ND	80	0.60	blind loop syndrome
**mean ± SD**	57.9 ± 21.4		20.6 ± 3.7		0.17 ± 0.02*	5.10 ± 1.08	2.23 ± 0.87	150.3 ± 57.0^+^	73.3 ± 36.7*	140.1 ± 65.0	0.73 ± 0.22	

*P < 0.05 vs. NS group,

^+^P < 0.01 vs. NS group.

Proteinuria; (-): ~9 mg/dl, (±): 10~29 mg/dl, (1+): 30~99 mg/dl, (2+): 100~299 mg/dl, (3+): 300~999 mg/dl, (4+): 1000 mg/dl~

Abbreviations. NS: nephrotic syndrome, M: male, F: female, BMI: body mass index, U-pro/cr: urinary protein-creatinine ratio, TP: serum total protein, Alb: serum albumin, T-cho: serum total cholesterol,

LDL-C: serum low-density lipoprotein cholesterol, TG: serum triglycerides, Cr: serum creatinine, ND: no data.

### Scintigraphy

All patients received a single intravenous dose of 740 MBq of ^99m^Tc-HSAD (Poolscinti, Nihon Medi-Physics, Tokyo, Japan), and static images (both anterior and posterior images) were obtained 24 hours after the administration of the radiopharmaceutical with the patient being kept in the supine position for eight minutes. The radiochemical purity level of 99mTc-HSAD was higher than 90% [6].

Scintigraphy was performed using a digital gamma camera (Symbia E, Siemens Japan K.K., Tokyo, Japan) with a rectangular field detector attached to a low-energy, high-resolution collimator. A 15% energy window centered over 140 keV (99mTc photopeak) with a matrix of 512 ×512 and magnification of 1.45x was used. We processed the images and analyzed the results using the Syngo MI Workplace (Siemens Japan K.K., Tokyo, Japan).

### Analytical methods (Assessments)

#### Method 1

**Fig 2:**
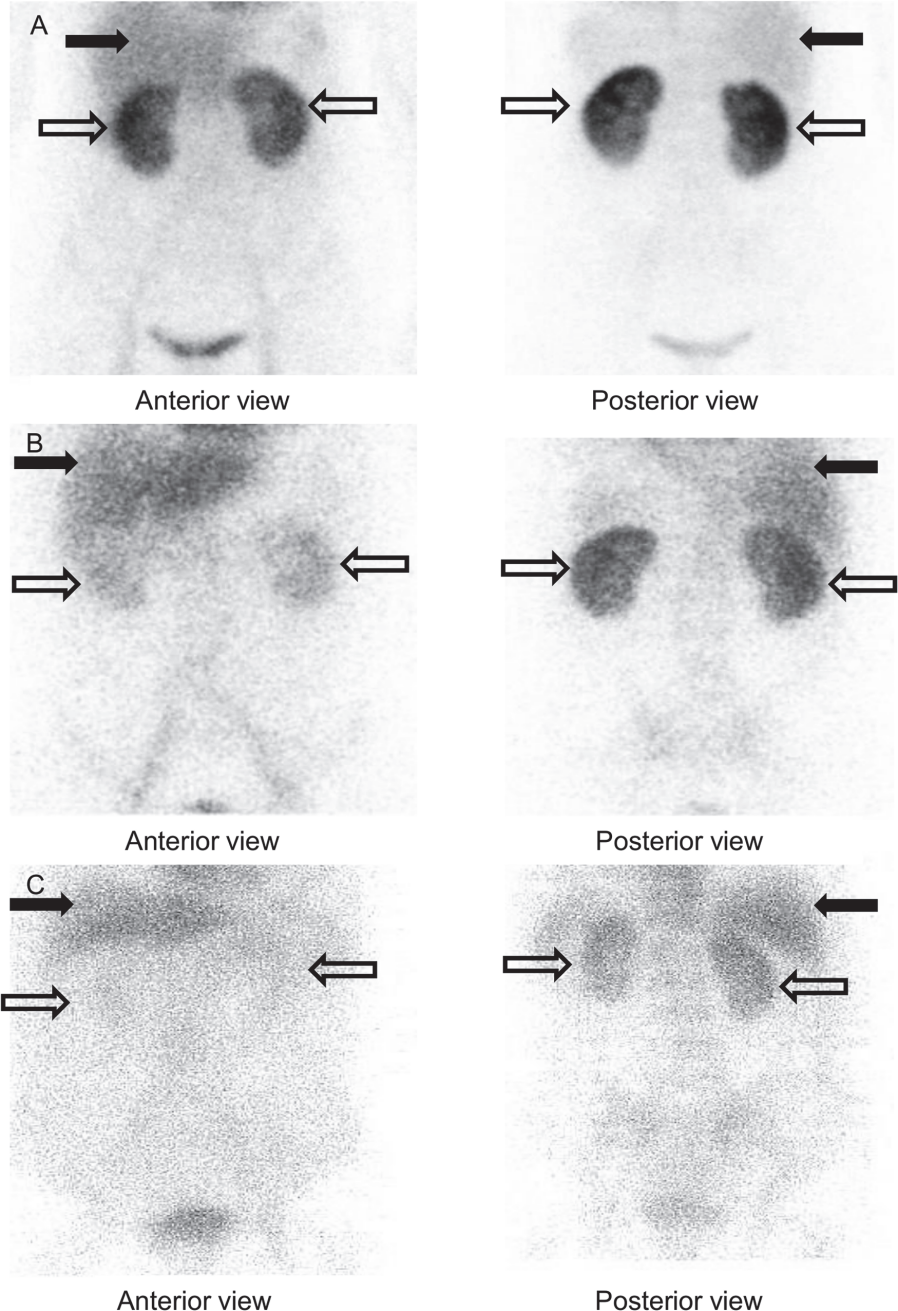
Definition of Dense Kidney. We defined the presence of a more intense uptake in the kidney (*white arrow*) than the liver (*black arrow*) on the anterior view 24 hours after ^99m^Tc-HSAD administration as Dense Kidney (+) (A), the same uptake in the liver and kidney as Dense Kidney (±) (B) and a more intense uptake in the liver than the kidney as Dense Kidney (-) (C).

In the qualitative analysis, we defined the presence of a more intense uptake in the kidney than the liver on the anterior view 24 hours after ^99m^Tc-HSAD administration as Dense Kidney (+) (Fig 2A), the same uptake in the liver and kidney as Dense Kidney (±) (Fig 2B) and a more intense uptake in the liver than the kidney as Dense Kidney (-) (Fig 2C) and compared the results between the two groups visually.

#### Method 2

Regions of interest in the right and left kidney and liver were designed on anterior and posterior images, respectively, in order to obtain the count per region 24 hours after ^99m^Tc-HSAD administration. The semi-quantitative analysis was performed by dividing the count per region in the right and left kidney by the count per region in the liver (kidney-liver ratio: K/L) and comparing the results between the two groups.

### Biochemical analysis

All biochemical assays were performed at the laboratory of Saga University Hospital, which complied with the International Federation of Clinical Chemistry and Laboratory Medicine. The urinary protein from spot urine samples was measured using the pyrogallol red method, and the urinary creatinine from spot urine was measured using an enzymatic method. Serological tests were done by the following methods: the total protein was determined by the Biuret reaction, albumin by the modified BCP assay, and the total cholesterol, low-density lipoprotein cholesterol, triglycerides and creatinine levels were determined by enzymatic methods. The time interval between the biochemical tests and imaging studies in all cases was less than 14 days.

### Statistical analysis

The statistical significance of differences between the groups was determined using Student's t-test or the Mann-Whitney U test. A two-sided P-value was considered to be significant for P values of < 0.05. The data are expressed as the mean ± SD, and K/L is also presented as geometric mean ± geometric SD in parentheses. All analyses were performed using the SPSS 22.0 (IBM Japan, Tokyo, Japan).

## Results

### Characteristics (Table 1)

The U-Pro/Cr (6.20 ± 2.34: 0.17 ± 0.02, P < 0.05) and serum total cholesterol (316.4 ± 113.7: 150.3 ± 57.0 mg/dL, P < 0.01) and serum low-density lipoprotein cholesterol (216.8 ± 99.8: 73.3 ± 36.7 mg/dL, P < 0.05) levels were significantly higher in the NS group than in the non-NS group, reflecting the clinical differences in pathophysiology between the two groups. There were no significant differences between the groups in the other parameters.

### Method 1 (Table 2)

In the NS group, nine of the ten patients (90%) had Dense Kidney (+), one patient (10%) had Dense Kidney (±) and no patients had Dense Kidney (-). In the non-NS group, no patients had Dense Kidney (+), five of the seven patients (71%) had Dense Kidney (±) and two patients (29%) had Dense Kidney (-), with significant differences between the groups (P < 0.001).

**Table 2:** Results of Method 1.

NS group (n = 10)	Dense Kidney	non-NS group (n = 7)	Dense Kidney
**Case 1**	(+)	**Case 11**	(-)
**Case 2**	(+)	**Case 12**	(±)
**Case 3**	(+)	**Case 13**	(-)
**Case 4**	(+)	**Case 14**	(±)
**Case 5**	(±)	**Case 15**	(±)
**Case 6**	(+)	**Case 16**	(±)
**Case 7**	(+)	**Case 17**	(±)
**Case 8**	(+)		
**Case 9**	(+)		
**Case 10**	(+)		
**Dense Kidney (+)**	n = 9 (90%)		n = 0 (0%)
**Dense Kidney (±)**	n = 1 (10%)		n = 5 (71%)
**Dense Kidney (-)**	n = 0 (0%)	** **	n = 2 (29%)

Abbreviations. NS: nephrotic syndrome.

The visual analysis clearly showed that the renal uptake of ^99m^Tc-HSAD was remarkably more intense in the NS group than in the non-NS group.

### Method 2

The semi-quantitative analysis confirmed the results of Method 1.

The liver is located ventral to the kidney anatomically; therefore, the K/L was lower on the anterior view than on the posterior view 24 hours after ^99m^Tc-HSAD administration.

**Fig 3:**
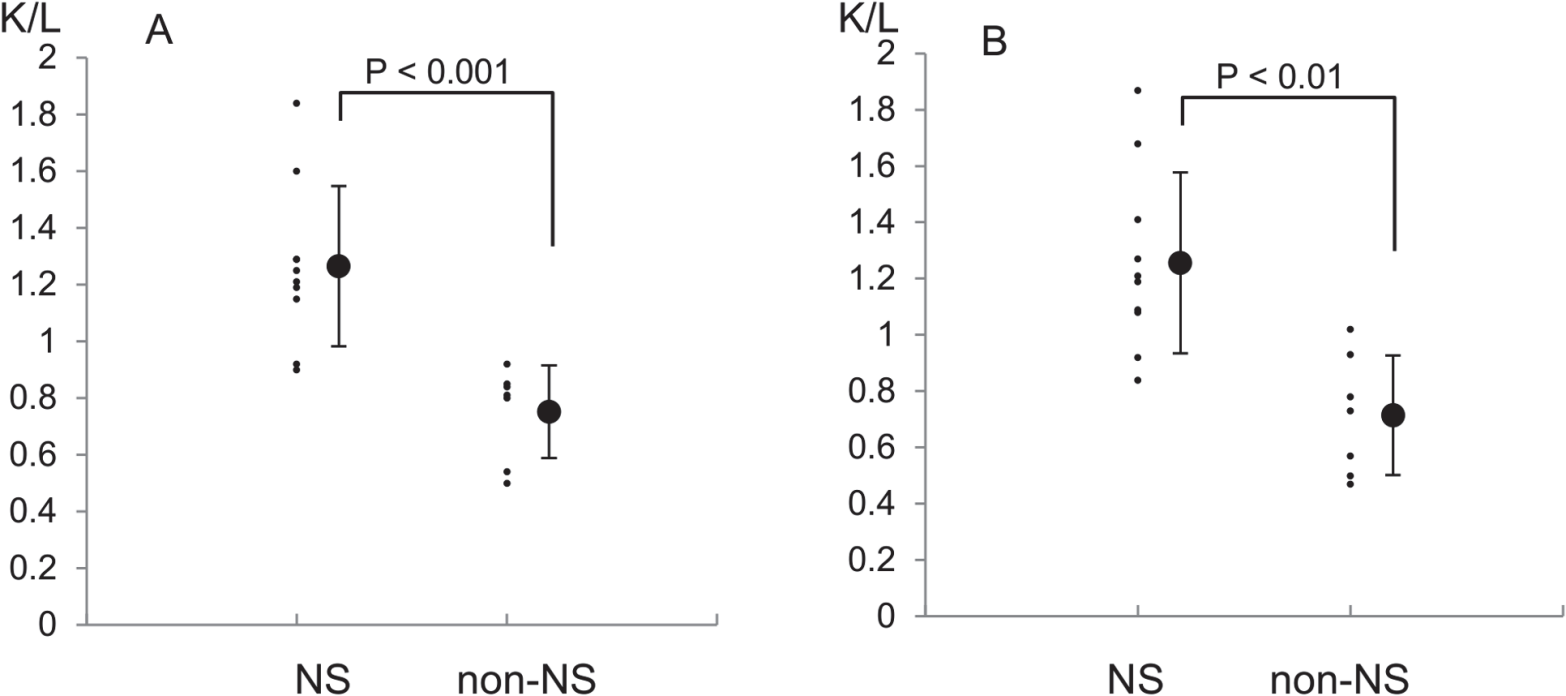
Quantification of the Tc-99m uptake in the nephrotic syndrome and non- nephrotic syndrome groups on the anterior view. A: right kidney, B: left kidney.

On the anterior view, the K/L in the right kidney was 1.26 ± 0.28 (1.24 ± 1.24) in the NS group and 0.75 ± 0.16 (0.73 ± 1.27) in the non-NS group, while that in the left kidney was 1.26 ± 0.32 (1.22 ± 1.28) in the NS group and 0.71 ± 0.21 (0.69 ± 1.35) in the non-NS group (Fig 3A and 3B). A comparison of the K/L on the anterior view showed significant differences between the groups (P < 0.001 for the right kidney and P < 0.01 for the left kidney).

**Fig 4:**
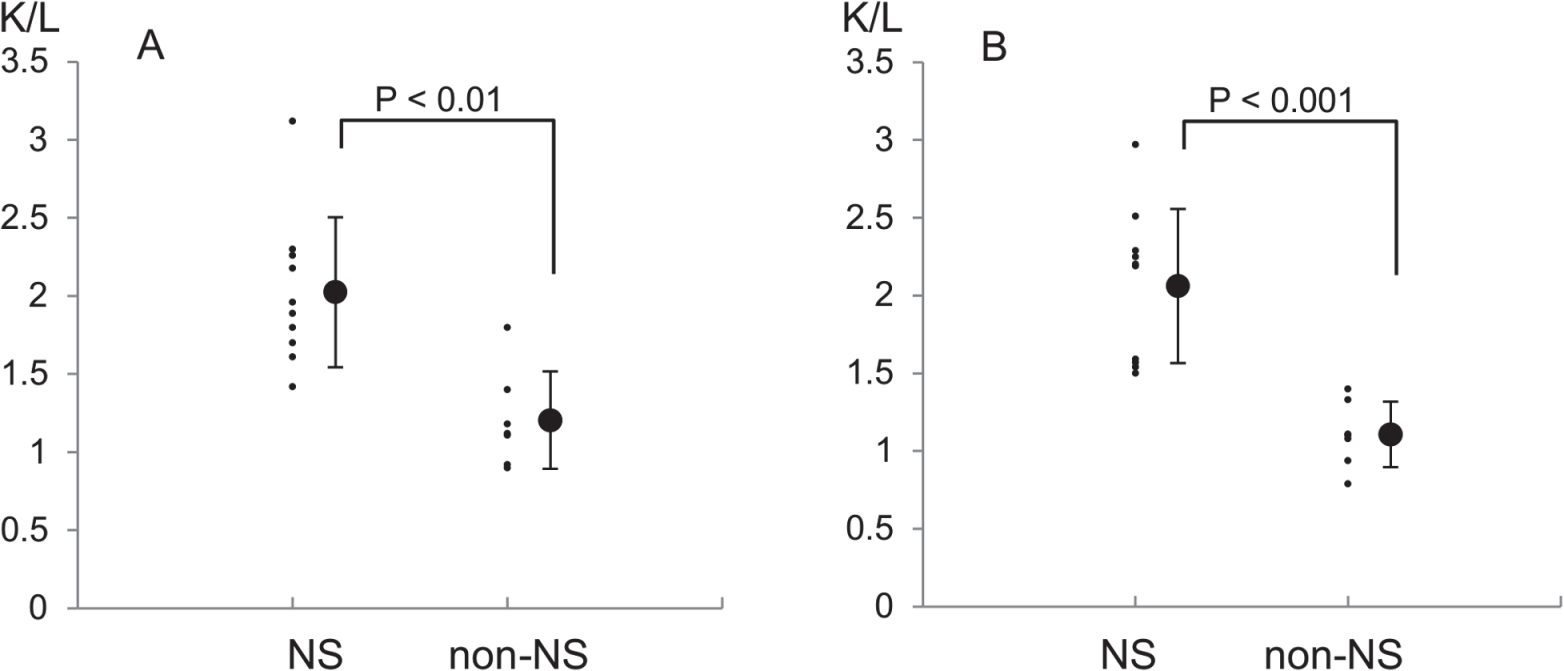
Quantification of the Tc-99m uptake in the nephrotic syndrome and non- nephrotic syndrome groups on the posterior view. A: right kidney, B: left kidney.

On the posterior view, the K/L in the right kidney was 2.02 ± 0.48 (1.98 ± 1.25) in the NS group and 1.20 ± 0.31 (1.17 ± 1.27) in the non-NS group, while that in the left kidney was 2.06 ± 0.49 (2.01 ± 1.27) in the NS group and 1.11 ± 0.21 (1.09 ± 1.22) in the non-NS group (Fig 4A and 4B). A comparison of the K/L on the posterior view showed significant differences between the groups (P < 0.01 for the right kidney and P < 0.001 for the left kidney). Therefore, the K/L was significantly higher in the NS group than in the non-NS group on every view.

## Discussion

NS is a pathophysiological condition in which a large quantity of proteinuria (more than 3.5 g/day) results in hypoproteinemia. However, the mechanism of hypoproteinemia in NS is unclear [7]. In patients with NS, massive protein leakage in the urine is the main cause [8,9], although many patients with nephrotic range proteinuria do not always develop hypoproteinemia. A previous study reported the findings of a group without hypoalbuminemia who exhibited proteinuria greater than 5 g/day in comparison with a group with similar proteinuria excretion but persistent hypoalbuminemia [10]. Another study reported that the serum protein levels are much lower in patients with NS than in those on peritoneal dialysis with protein leakage into peritoneal dialysis fluid at the same level as NS [11].

Analyses of primitive urine gathered according to the micropuncture method have shown that only a very small amount of albumin leaks through the glomerulus in healthy individuals [12], while almost 100% of albumin in primitive urine is reabsorbed at the proximal tubule epithelium (PTE); therefore, almost no albumin is included in the final urine. Recently, a method for visualizing the dynamics of proteins in living bodies was developed, and the results of examinations of albumin metabolism dynamics in the kidneys using fluorescence-labeled albumin and two-photon microscopy were reported. Based on the findings, even in the normal state, albumin leaking from the glomerulus is taken into the PTE via endocytosis through receptors and collected in the blood [13]. It is difficult to explain the occurrence of hypoalbuminemia based on only the quantity of albumin lost in the urine, and the catabolic reaction that occurs when serum albumin is reabsorbed via the PTE following glomerular filtration is thought to be an important causative factor of hypoalbuminemia in patients with NS [14–16]. As mentioned above, proteins that leak through the glomeruli combine with megalin and cubilin in the PTE and are taken into cells via endocytosis and reabsorbed in the blood after being disintegrated into amino acids in endosomes by lysosomal enzymes [17]. In addition, it is possible that the albumin taken into cells is collected via transcytosis, and studies visualizing and quantifying the albumin degradation process in the kidneys using an albumin reagent (dye-quenched albumin) that emits fluorescence when disintegrated by protein-degrading enzymes were recently reported. According to the results, after albumin is taken into the PTE, vesicular trafficking is carried out in the cell. However, approximately 40% of proteins are disintegrated immediately [18]. The failure of the recovery mechanism in patients with NS has received significant attention, as such failure may induce a high level of proteinuria.

Hepatic albumin synthesis increases in response to albumin loss. This effect is mediated by an increase in the hepatic albumin gene expression stimulated in part by a physical factor, low oncotic pressure, which is regulated by the direct effects of low oncotic pressure in hepatocytes [19,20]. Hypoalbuminemia may also stimulate the release of an as yet unidentified circulating factor that contributes to elevation in hepatic albumin synthesis [21]. On the other hand, the active direct suppression of hepatic albumin synthesis by lymphokines, for example, tumor necrosis factor and interleukin-1, has been described in NS [22]. It eventually remains unclear why, in patients excreting 4 or 6 g of protein per day, the liver is usually unable to sufficiently increase albumin synthesis to normalize the plasma albumin concentration.

Furthermore, in NS, protein leakage due to gastrointestinal and stromal edema resulting from capillary hyperpermeability is considered to be a causative factor of hypoproteinemia [23].

99mTc-HSAD is human serum albumin (HSA) labelled by 99mTc via diethylenetriamine pentaacetic acid (DTPA) due to its strong affinity for 99mTc. Therefore, it has higher label and blood retention rates, superior stability *in vivo* and can be used to obtain a clear scintigram against a low background compared to ^99m^Tc-Human Serum Albumin (^99m^Tc-HSA) or ^99m^Tc-Red Blood Cell (^99m^Tc-RBC) [24,25]. Hence, it is useful for the diagnosis and follow-up of the hemodynamics of various organs in patients with protein-losing gastroenteropathy and vascular diseases, such as heart disease, major vascular disease, peripheral circulatory diseases and cerebral vascular disease [26–30].

As mentioned above, when healthy adults are intravenously given ^99m^Tc-HSAD for protein leakage scintigraphy, the same dynamics are observed as in albumin metabolism, and a previous study showed that the organ radioactivity (%ID) of the liver and kidneys after 24 hours is equal, at approximately 10% in humans and 7% in rats [5,6]. In Method 1 of this study, because the liver is located ventral to the kidney anatomically, we assessed the differences between groups on the anterior, not posterior view, in order to visually confirm that the renal uptake was more intense than that in the liver. Both Method 1 and Method 2 showed that the renal uptake of ^99m^Tc-HSAD was clearly more intense in the NS group than in the non-NS group, independent of the serum albumin concentration (Table 1). On the other hand, there was no correlation between serum albumin and degree of proteinuria (U-Pro/Cr) in nine patients with Dense Kidney (Pearson’s correlation coefficient -0.12). Therefore, it is possible that Dense Kidney reflects the reabsorption of albumin at the proximal tubule and would be helpful to interpret hypoalbuminemia in NS cases with limited urine protein excretion.

It is also possible that the above-mentioned two previously reported cases were in the pre-stage of NS and a prolonged stage of healing, respectively. However, nephrologists occasionally encounter patients without nephrotic range proteinuria who exhibit hypoproteinemia in whom the cause of hypoproteinemia is thought to be similar to the pathophysiology of NS. Because NS induces systemic edema, susceptibility to infection due to decreased immunoglobulin production and thrombogenesis resulting from hypercoagulability, early diagnosis and proper treatment are necessary. However, steroid and immunosuppressive therapy carry risks of side effects. Therefore, treatment based on an easy diagnosis is also dangerous.

In conclusion, our results suggest that, in patients who do not exhibit a large enough quantity of urine protein to cause NS and scintigraphy indicates Dense Kidney, the pathophysiology involves hyperfunction of urine protein reabsorption at the proximal tubule with massive leakage from the glomeruli (i.e., a similar pathophysiology to NS). Such findings also aid in the diagnosis of cases in which it is difficult to perform a renal biopsy. We believe that the detection of Dense Kidney using the method described in this study will therefore be a useful diagnostic modality for diagnosing nephrotic syndrome. In the same manner, positive renal scan using gallium-67 citrate scintigraphy has now become a widely accepted examination for diagnosing acute tubulointerstitial nephritis [31–33].

Because the evaluation in Method 1 lacked objectivity, we measured the relative contrast ratio of the kidney to the liver in Method 2 and were able to identify significant differences more objectively. However, when used as a supporting examination, Method 1 is a simple and easy tool that is clinically available. Therefore, it is necessary to accumulate more cases including repeating studies during remission of NS in order to assess the usefulness of this method for diagnosis. However, we should be more cautious in the indication for this type of studies and reserve for specific situations of unexplained hypoalbuminemia because the patient may receive a radiation dose of approximately 6mSv by this examination.

## Conclusions

The present results suggest that the detection of Dense Kidney 24 hours after ^99m^Tc-HSAD administration for protein leakage scintigraphy reflects the pathophysiology of hyperfunction of urine protein reabsorption at the proximal tubule with massive leakage from the glomeruli. This diagnostic modality is therefore expected to be an effective examination for diagnosing patients without nephrotic range proteinuria which has a pathophysiology similar to that of NS.
